# Clinical Evaluation of Tumour Marker Combinations in the Differential Diagnosis of Benign and Malignant Liver Disease

**DOI:** 10.1155/1991/23874

**Published:** 1991

**Authors:** M. E. Lucarotti, N. D. Rothnie, S. B. Kelly, M. J. Hershman, C. Johanssen, O. Nilsson, L. Lindholm, C. B. Wood, R. C. N. Williamson, N. A. Habib

**Affiliations:** 1 University Department of Surgery Bristol Royal Infirmary London UK; 2 Department of Surgery Royal Postgraduate Medical School London UK; 3 Department of Immunology Gotenberg University Gotenberg Sweden; 4 University Department of Surgery Bristol Royal Infirmary Bristol BS2 8HW UK

## Abstract

CEA, CA19-9 and CA50 are tumour associated antigens defined by monoclonal antibodies which have been raised against adenocarcinoma cell lines. The aim of this study was to determine whether their combined use could improve diagnostic accuracy in patients with primary and secondary liver tumours. An immunoradiometric assay was used for the detection of CEA and CA19-9 and the Delfia system for CA50. Serum was collected from 65 normal subjects, 40 with hepatobiliary carcinoma (26 primary, 14 secondary) and 17 with benign hepatobiliary disease. The cut-off levels were calculated as the mean of the control group plus 2 standard deviations. All three antibodies contributed to improving the correct classification of secondary liver tumours (multivariant discriminant analysis p<0.05), but only CA19-9 and CA50 contributed to the diagnosis of primary liver tumours (multivariant analysis p<0.05). The diagnostic accuracy versus benign disease was 81% for primary carcinoma and 91% for secondary carcinoma. Combined use of CEA, CA19-9 and CA50 helps to differentiate benign from malignant hepatobiliary disease.


*HPB Surgery*, 1991, Vol. 5, pp. 23–28
Reprints available directly from the publisher
Photocopying permitted by license only

© 1991 Harwood Academic Publishers GmbH
Printed in the United Kingdom

### CLINICAL EVALUATION OF TUMOUR MARKER COMBINATIONS IN THE DIFFERENTIAL DIAGNOSIS OF BENIGN AND MALIGNANT LIVER DISEASE

M.E. LUCAROTTI,† N.D. ROTHNIE,† S.B. KELLY,† M.J. HERSHMAN,* C. JOHANSSEN,‡ O. NILSSON,‡ L. LINDHOLM,‡ C.B. WOOD,* R.C.N. WILLIAMSON,* N.A. HABIB†

*†University Department of Surgery, Bristol Royal Infirmary, *Department of Surgery, Royal Postgraduate Medical School, London, ‡Department of Immunology, Gotenberg University, Gotenberg, Sweden*

*(Received 19 February 1991)*

CEA, CA19-9 and CA50 are tumour associated antigens defined by monoclonal antibodies which have been raised against adenocarcinoma cell lines. The aim of this study was to determine whether their combined use could improve diagnostic accuracy in patients with primary and secondary liver tumours. An immunoradiometric assay was used for the detection of CEA and CA19-9 and the Delfia system for CA50. Serum was collected from 65 normal subjects, 40 with hepatobiliary carcinoma (26 primary, 14 secondary) and 17 with benign hepatobiliary disease. The cut-off levels were calculated as the mean of the control group plus 2 standard deviations. All three antibodies contributed to improving the correct classification of secondary liver tumours (multivariant discriminant analysis $p<0.05$), but only CA19-9 and CA50 contributed to the diagnosis of primary liver tumours (multivariant analysis $p<0.05$). The diagnostic accuracy versus benign disease was 81% for primary carcinoma and 91% for secondary carcinoma. Combined use of CEA, CA19-9 and CA50 helps to differentiate benign from malignant hepatobiliary disease.

KEY WORDS: Tumour markers, liver tumours, diagnosis

### INTRODUCTION

In the last three decades hepatobiliary surgery has progressed to become a distinct sub-speciality, with a mortality rate for hepatic resection below 5%[1,2]. Hepatectomy may be suitable for primary malignant tumours (hepatocellular carcinoma, cholangiocarcinoma), but in the western world these are outnumbered by liver metastases, 70% of which originate from colorectal primary lesions[3]. Since most patients with colorectal secondaries are asymptomatic at first, a sensitive test is required to diagnose them early enough for surgical treatment still to be feasible; determination of high levels of carcino-embryonic antigen (CEA) in the serum is one such possibility[4]. At present resection of colorectal liver metases is only possible in 30–40% of patients,[5,6] but up to 25% of these may be expected to survive 5 years[1].

Address correspondence to: Miss M.E. Lucarotti, University Department of Surgery, Bristol Royal Infirmary, Bristol, BS2 8HW

Among primary hepatic malignancies, hepatocellular carcinoma (HCC) is increasing in incidence,[7] but patients tend to present at a late stage with irresectable lesions. For small lesions discovered even before the onset of symptoms, for example by ultrasound scan or a raised alpha-feto protein (AFP) level, resection could be curative. The other common primary liver tumour, cholangiocarcinoma, has no associated tumour marker currently available[8]. Resection is only appropriate for 10–30% of cholangiocarcinomas, but when possible it is the most effective treatment[8,9]. Tumour markers may offer an opportunity for the detection of early disease.

CEA, CA 19-9 and CA 50 assays are based on monoclonal antibodies raised against human colorectal carcinoma cell lines[10,11]. Elevated serum levels have been found in patients with gastrointestinal and other malignancies[12,13,14]. The present study assesses the sensitivity and specificity of these three tumour markers in the differential diagnoses of benign and malignant liver disease.

### PATIENTS AND METHODS

Single serum samples were collected from 65 normal healthy control subjects, 40 patients with hepatobiliary carcinoma and 17 patients with benign hepatobiliary disease (Table 1). Serum was stored at –20°C until analysis. The primary hepatobiliary cancer group included 13 patients with cholangiocarcinoma, 12 with hepatocellular carcinoma and one with carcinoma of the gall bladder. The metastatic group contained 12 colonic and 2 gastric primaries.

**Table 1** Details of patients

| *Patients* | *n* | *Age (yr)* *Median* | *Range* |
|---|---|---|---|
| Controls | 65 | 42 | 8–98 |
| Benign hepatobiliary disease[a] | 17 | 58 | 12–74 |
| Primary liver malignancy | 26 | 47 | 17–71 |
| Secondary liver malignancy | 14 | 53 | 17–78 |

[a] comprising haemangioma (3), hydatid disease (3), sclerosing cholangitis (2), viral hepatitis (2) and one each of simple cyst, portal hypertension, primary biliary cirrhosis, biliary stricture, choledocholithiasis, cholangitis and hepatic sarcoid.

A commercial kit was used for the detection of CEA and CA 19-9. ELSA-CEA is a solid-phase two-site immunoradiometric assay specific for CEA. Three monoclonal antibodies are prepared against sterically remote antigenic sites on the CEA molecule: two of them are coated on the ELSA solid phase, while the third one is radiolabelled with 125 iodine and used as a marker. Following the formation of the coated antibody-antigen-iodinated antibody sandwich, the unbound tracer is removed by washing. The radioactivity bound to the ELSA is proportional to the concentration of CEA present in the sample.

CIS ELSA CA 19-9™ is a solid phase (ELSA) immunoradiometric assay specific for CA 19-9. ELSA coated with mouse monoclonal antibody to CA 19-9™ is incubated with the sample. CA 19-9™ then binds the ELSA. The same monoclonal anti CA 19-9™ then binds with ELSA. Unbound tracer is removed by washing. The radioactivity bound to ELSA is proportional to the concentration of CA 19-9 in the sample.

CA50 was detected by the disassociation enhanced lanthamide fluorimmunoassay (DELFIA) technique (Stena Diagnostics, Sweden). The Delfia technique is a solid-phase immunofluorometric assay based on a direct sandwich technique.

For each assay a cut-off level was calculated according to the mean value of the control group plus two standard deviations as follows CA19-9 46 u/ml, CEA7 μg/ml, CA50 21 u/ml. Data obtained were studied with multivariant discriminant analysis to produce a model (based on known normal and abnormal values) which is a linear combination giving the widest discrimination between tested normal and abnormal subjects.

### RESULTS

Table 2 shows the number of patients and controls with 'positive' tumour markers. Of 40 patients with hepatobiliary cancer 18 (45%) had a CA 19-9 level above 46 u/ml,, 22 (55%) had a CA50 level above 21 u/ml and 15 (48%) had a CEA level above 7 μg/ml. To improve specificity we used multivariant discriminant analysis of the combination of antibodies. The analysis showed a positive result with 53% of the whole malignant group, none of the benign group and 8% of the control group. All three antibodies contributed to the diagnostic accuracy for the whole malignant group (80%) and for secondary tumours (91%) but only CA 19-9 and CA50 contributed to improved diagnostic accuracy in the primary group alone (81%) (Figure 1, Table 4). Further breakdown of the primary malignant group shows that CEA was never positive in patients with hepatocellular carcinoma (Table 3).

**Table 2** Number of patients and controls with 'positive' tumour markers (i.e., values above cut-off level)

| | *n* | *CA19-9* | *CA50* | *CEA* | *Combination of all 3 markers*[a] |
|---|---|---|---|---|---|
| Controls | 65 | 6 | 7 | 5 | 5 |
| Benign | 17 | 0 | 4 | 0 | 0 |
| Primary | 26 | 12 | 13 | 5 | 11 |
| Secondary | 14 | 6 | 9 | 10 | 10 |
| Primary and Secondary | 40 | 18 | 22 | 15 | 21 |

[a] i.e. Patients positive with multivariant discriminant analysis.

**Table 3** Percentage positive in patients with primary and secondary liver malignancies

| | *CA19-9* | *CA50* | *CEA* | *Combination of all three markers* |
|---|---|---|---|---|
| Cholangiocarcinoma (n = 14) | 62 | 62 | 38 | 54 |
| Hepatocellular carcinoma (n = 12) | 25 | 42 | 0 | 25 |
| Secondary carcinoma (n = 14) | 43 | 64 | 71 | 71 |

**Table 4** Sensitivity and specificity of markers in primary and secondary liver malignancy

*Primary liver malignancy*

| | *CA19-9* | *CA50* | *CEA* | *Combination of all three markers* |
|---|---|---|---|---|
| Sensitivity (%) | 46 | 50 | 19 | 42 |
| Specificity (%) | 93 | 87 | 94 | 94 |
| Diagnostic accuracy (%) | 81 | 78 | 76 | 81 |

*Secondary liver malignancy*

| | *CA19-9* | *CA50* | *CEA* | *Combination of all three markers* |
|---|---|---|---|---|
| Sensitivity (%) | 43 | 64 | 71 | 71 |
| Specificity (%) | 93 | 87 | 94 | 94 |
| Diagnostic accuracy (%) | 85 | 83 | 91 | 91 |

*Primary and secondary liver malignancy*

| | *CA19-9* | *CA50* | *CEA* | *Combination of all three markers* |
|---|---|---|---|---|
| Sensitivity (%) | 45 | 55 | 38 | 53 |
| Specificity (%) | 93 | 87 | 94 | 94 |
| Diagnostic accuracy (%) | 77 | 76 | 75 | 80 |

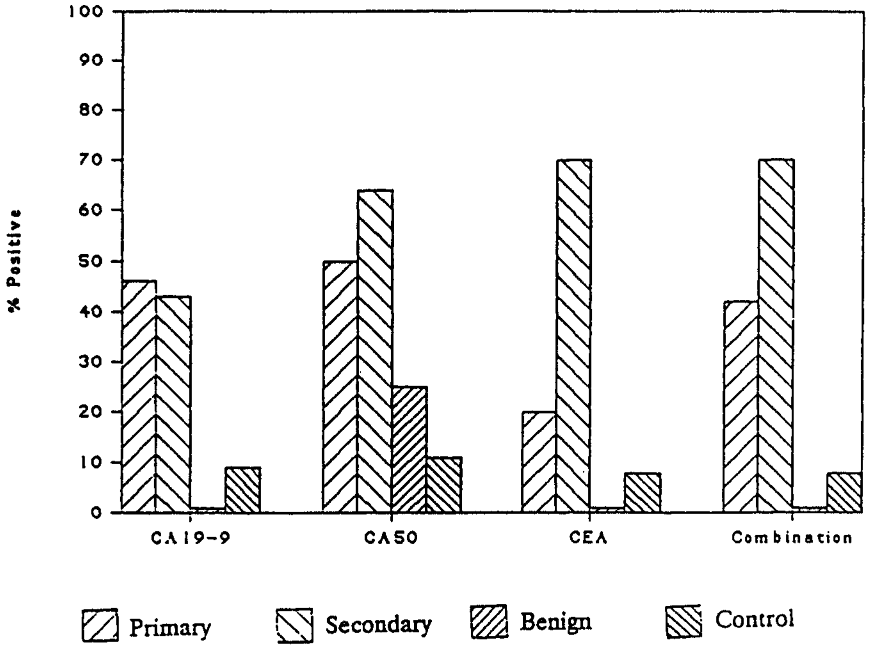

**Figure 1** Percentage of patients with positive tumour markers, using single and combined antibodies.

### DISCUSSION

In this study the combination of antibodies was positive in 11 of 26 patients (42%) with primary liver tumours and 10 of 14 patients (71%) with secondary liver tumours, but in none of 17 patients with benign hepatobiliary diseases and only 5 of 65 (8%) normal controls. There was a slight gain in diagnostic accuracy, largely because of greater specificity, when the combination antibodies was employed over the single use of any one test. This combination technique could therefore have a useful additive value to existing diagnostic methods in the screening of high risk groups, for example asymptomatic patients with liver cirrhosis, or those with potentially curative resection of colorectal carcinoma. Such a policy might help in the early detection of liver tumours at an asymptomatic stage where surgical resection could still be possible.

Although surgical removal remains the only treatment with any hope of long-term survival in patients with liver cancer, the extent of the disease usually precludes hepatic resection by the time symptoms become established[15]. Bismuth *et al.*[16] reported an incidence of resection of 10% in symptomatic patients with hepatocellular carcinoma with liver cirrhosis, and only one of 35 patients survived more than two years. On the other hand, Yu, Tang and Zhou[17] reported a 60% five-year survival in patients with liver cirrhosis undergoing a comprehensive screening programme for HCC at an asymptomatic stage. Thus screening of high risk groups should be implemented, and the use of a tumour marker combination complemented by AFP measurement and ultrasonography seems the best method currently available.

### *References*

1. Adson, M.A., van Heerden, J.A., Adson, M.H., Wagner, J.S. and Illstrup, D.M. (1984) Resection of hepatic metastases from colorectal cancer. *Archives of Surgery*, **119**, 647–651
2. Nordlinger, B., De Sena, G., Szawlowski, A., Orea Martinez, J.G., Park, R., Malafosse, M., *et al.* (1983) Resection chirurgicale des metastases hepatiques des cancers du colon et du rectum. *Gastroenterologie Clinique et Biologique*, **7**, 240–243
3. Rougier, P., Elias, D., Lasser, P., *et al.* (1985) Circomstances de decouverte et facteurs pronostiques de metastases hepatiques. In: *Actualites carcinologiques*, pp. 81–88. Paris: Masson
4. Staab, H.J., Anderer, F.A., Stumpf, E., Hornung, A. and Keininger, G. (1985) Eight-four potential second-look operations based on sequential carcinoembryonic antigen determinations and clinical investigations in patients with recurrent gastrointestinal cancer. *American Journal of Surgery*, **149**, 198–204
5. August, D.A., Sugarbaker, P.H., Ottow, R.T., Gianola, F.J. and Scheinder, P.D. (1985) Hepatic resection of colorectal metastases. Influence of clinical factors and adjuvant intraperitoneal 5 — fluorouracil via Tenckhoff catheter on survival. *Annals of Surgery*, **201**, 210–218
6. Fortner, E.R., Silva, J.S., Cox, E.G., Maclean, B.J. (1984) Multivariate analysis of a personal series of 247 consecutive patients with liver metastases from colorectal cancer.. I. Treatment by hepatic resection. *Annals of Surgery*, **199**, 306–316
7. Brocheriou, C., Auriol, M., Ajebo, M., Chomette, G. (1974) Les hepatomes. Denombrement necropsies. *Annales de Medecine Interne*, **126**, 265–268
8. Cooper, M.M. (1986) Neoplasms of the biliary tree. *Current Opinion in Gastroenterology*, **2**, 729–734
9. Langer, J.C., Langer, B., Taylor, B.R., Zeldin, R., Cummings, B. (1985) Carcinoma of the extrahepatic bile ducts: Results of an aggressive surgical approach. *Surgery*, **98**, 752–759
10. Koprowski, H., Steplewski, Z., Mitchell, K., Herlyn, M., Herlyn, D. and Fuhrer, P. (1979) Colorectal carcinoma antigens detected by hybridoma antibodies. *Somatic Cell and Molecular Genetics*, **5**, 957–962

11. Lindholm, L., Holmgren, J., Svennerholm, L. *et al.* (1983) Monoclonal antibodies against gastrointestinal tumour-associated antigens isolated as monosialogangliosides. *International Archives of Allergy and Applied Immunology,* **71**, 178–181
12. Herlyn, M., Sears, H.F., Steplewski, Z., Koprowski, H. (1982) Monoclonal antibody detection of a circulating tumour-associated antigen. 1. Presence of antigen in sera of patients with colorectal, gastric and pancreatic carcinoma. *Journal of Clinical Immunology,* **2**, 135–140
13. Holmgren, J., Lindholm, L., Persson, B. *et al.* (1984) Detection by monoclonal antibody of carbohydrate antigen CA50 in serum of patients with carcinoma. *British Medical Journal,* **288**, 1479–1482
14. Moore, T.L., Kupchik, H.K., Marcon, N., Samcheck, N. (1971) Carcinoembryonic antigen assay in cancer of the colon and pancreas and other digestive tract disorders. *American Journal of Digestive Disorders,* **16**, 1–7
15. Okuda, K., Obata, H., Nakajima, Y., Ohtsuki, T., Okasaki, N., Ohnishi, K. (1984) Prognosis of primary hepatocellular carcinoma. *Hepatology,* **4**, 35–65
16. Bismuth, H., Houssin, D., Ornowski, J., Meriggi, F. (1986) Liver resections in cirrhotic patients: A Western experience. *World Journal of Surgery,* **10**, 311–317
17. Yu, Y., Tang, Z.Y., Zhou, X. (1980) Experience in resection of small hepatocellular carcinoma. *Chinese Medical Journal,* **93**, 491–496

*(Accepted by S. Bengmark 23 April 1991)*

